# New Approach for Using of *Mentha longifolia* L. and *Citrus reticulata* L. Essential Oils as Wood-Biofungicides: GC-MS, SEM, and MNDO Quantum Chemical Studies

**DOI:** 10.3390/ma14061361

**Published:** 2021-03-11

**Authors:** Hayssam M. Ali, Wael A. A. Abo Elgat, Mervat EL-Hefny, Mohamed Z. M. Salem, Ayman S. Taha, Dunia A. Al Farraj, Mohamed S. Elshikh, Ashraf A. Hatamleh, Eslam M. Abdel-Salam

**Affiliations:** 1 Botany and Microbiology Department, College of Science, King Saud University, P.O. Box 2455, Riyadh 11451, Saudi Arabia; hayhassan@ksu.edu.sa (H.M.A.); dfarraj@ksu.edu.sa (D.A.A.F.); melshikh@ksu.edu.sa (M.S.E.); ahatamleh@ksu.edu.sa (A.A.H.); 436108002@student.ksu.edu.sa (E.M.A.-S.); 2 Agriculture Research Center, Timber Trees Research Department, Sabahia Horticulture Research Station, Horticulture Research Institute, Alexandria 21526, Egypt; 3 Restoration Department, High Institute of Tourism, Hotel Management and Restoration, Abukir, Alexandria 21526, Egypt; watsat20@yahoo.com; 4 Department of Floriculture, Ornamental Horticulture and Garden Design, Faculty of Agriculture (El-Shatby), Alexandria University, Alexandria 21545, Egypt; mervat.mohamed@alexu.edu.eg; 5 Forestry and Wood Technology Department, Faculty of Agriculture (EL-Shatby), Alexandria University, Alexandria 21545, Egypt; 6 Conservation Department, Faculty of Archaeology, Aswan University, Aswan 81528, Egypt; aymansalahtaha82@yahoo.com

**Keywords:** antifungal activity, essential oils, mass spectrometry, *Mentha longifolia*, *Citrus reticulata*, wood-biofungicide, MNDO quantum, fungi

## Abstract

Background: Fungi growing on wood cause deterioration of stored food materials or discoloration of the wood itself, and the search for new and safe bioagents is recently needed. Methods: Essential oils (EOs) from aerial parts from *Mentha longifolia* L. and *Citrus reticulata* L., analyzed by gas chromatography-mass spectrometry (GC-MS), were tested for their antifungal activity by the vapor method against four common fungi, *Aspergillus flavus*, *A. niger*, *A. fumigatus*, and *Fusarium culmorum*, and confirmed by SEM examination as the oils applied on wood samples. Results: The most abundant compounds identified in the EO from *M. longifolia* were menthone and eucalyptol; in *C. reticulata* EO, they were β-caryophyllene, β-caryophyllene oxide, and β-elemene. EOs from *M. longifolia* and *C. reticulata*, at 500 and 250 µL/mL, showed potent antifungal activity against *A. flavus* and *A. fumigatus*, with 100% fungal mycelial inhibition growth (FMIG). *C. reticulata* and *M. longifolia* EOs, at 125 µL/mL, observed FMIG values of 98% and 95%, respectively, against *A. fumigatus*. *M. longifolia* EO, at 500 and 250 µL/mL, showed potent activity against *A. niger*, with 100% FMIG. *F. culmorum* completely inhibited (100% FMIG) EOs from *M. longifolia* and *C. reticulata* applied at 500 µL/mL. *Pinus roxburghii* Sarg. Wood, treated with *M. longifolia* at 125 µL/mL, showed inhibition zone values of 7.33 and 21.33 mm against *A. flavus* and *A. niger*, respectively. Conclusions: Both oils possessed good wood-biofungicide activity with the vapor method, as clearly shown by the SEM examination. These activities suggest their possible use as natural wood preservatives.

## 1. Introduction

Fungi such as *Aspergillus niger*, *A. flavus*, *A. fumigatus*, *Alternaria tenuissima*, *Colletotrichum gloeosporioides*, *Fusarium culmorum*, *Penicillium chrysogenum*, *Rhizoctonia solani*, and *Trichoderma harzianum* are capable of growing upon a wide range of organic substrates of wood, lignocellulosic materials, and food, which can lead to the deterioration of stored food materials or the discoloration of wood or paper substances [1,2,3,4,5,6,7,8,9,10,11]. Essential oils (EOs)—aromatic substances—can be obtained from different plant parts such as leaves, flowers, seeds, fruits, bark, wood, and roots by extraction using steam or hydrodistillation. Some plant EOs have recently been proven to be successful as ecofriendly biocontrol agents, with antibacterial, antifungal, antioxidant, insecticidal, and antiviral properties [12,13,14,15,16,17,18,19,20,21], and have potential uses as natural additives and wood-biofungicides [2,3,4,6,7] and in the food industry, [22,23,24,25].

*Mentha longifolia* L. (or *M. lavandulacea* Willd. or *M. sylvestris* L.) has multipurpose use due to antimicrobial, antioxidant, and insecticidal activities [26,27]. Piperitone oxide (63.58%) and 1,8-cineole (12.03%)—oxygenated monoterpenes—were found as the main compounds in the EO of *M. longifolia* [27], with strong antibacterial activity against *Escherichia coli, Pseudomonas aeruginosa*, and *Salmonella enterica. cis*-Piperitone epoxide, piperitenone oxide and pulegone were the main components in *M. longifolia* ssp. *longifolia*, and the essential oil showed strong antimicrobial activity against 30 microorganisms [26]. Carvone, limonene, 1,6-dihydrocarveol, 1,8-cineole, *trans*-dihydrocarvone, β-bourbonene, germacrene D, β-caryophyllene, and bicyclosesquiphellandrene were found as the main compounds from *M. longifolia* EO collected from five regions of Saudi Arabia [28]. *M. longifolia* oils from different regions of South Africa showed good antibacterial activity against *S. aureus* and *E. coli* [29].

In fact, EOs from *M. longifolia* have shown widely variable antimicrobials against *S. aureus*, *Salmonella typhimurium*, *E. coli*, *F. oxysporum*, *A. flavus*, *A. niger*, *Microsporum canis*, *Mucor ramamnianus*, *Salmonella enteritidis* and *Klebsiella pneumonia* [30,31,32]. In addition, EOs from species of *Mentha* including *M. longifolia* exhibited significant antimicrobial activity against some bacterial and funal strains [33]. Furthermore, the EO from the dried herb showed potent antibacterial activity against *Bacillus subtilis*, *Micrococcus luteus* and *Enterococcus faecalis* [34]. Very strong antibacterial activity against *E. coli*, *Shigella sonei* and *Micrococcus flavus* and significant fungistatic activity against *Trichophyton tonsurans* and *Candida albicans* were found from the application of three Mentha species EO, including *M. longifolia* [35]. The EO of *M. longifolia* was observed to have higher antibacterial and antifungal activities than tested commercial substances [35]. The main compounds of *M. Longifolia* EO were piperitenone oxide (70%), piperitenone (18.7%) and 1,8-cineole (2.2%), and this oil suppressed the growth of *A. flavus* [36]. *M. longifolia* EO had significant antifungal and antioxidant activities; the main compounds were *trans*-dihydrocarvone (23.64%) and piperitone (17.33%) [31]. Recently, leaf EO, with piperitone and eucalyptol as the main compounds, showed potent antibacterial activity against *B. subtilis*, *S. aureus*, *P. aeruginosa* and *E. coli* [37].

*Citrus reticulata* L., belonging to the Rutaceae family, is an important fruit citrus-bearing plant with an excellent source of EO [38]. Peels of *C. reticulata* Blanco, with its main compounds of tangeretin, nobiletin, 5-demethylnobiletin, tetramethyl-o-scutellarein, tetramethyl-o-isoscutellarein, pentamethoxyflavone, and sinensetin, was observed to cause potent growth inhibition of *A. niger* [39]. Limonene and other compounds were found to be the major compounds in the EOs extracted from peels, in addition to other constituents such as sabinene, linalool, γ-terpinene, and methyl *N*-methylanthranilate, which were identified in leaf EO [40]. Generally, the EO from *C. reticulata* is regarded as safe, with excellent antifungal, antibacterial, and antioxidative properties and usefulness in food and medicine [41]. β-caryophyllene oxide, the epoxide derivative from β-caryophyllene, is a component of many EOs, especially *C. reticulata* [42].

Therefore, the aim of the present study is to evaluate the bioactivity of essential oils from *M. longifolia* aerial parts and *C. reticulata* leaves against some common molds and to identify the chemical composition of these oils using gas chromatography-mass spectrometry (GC-MS), with semiempirical calculations of molecules for the main compounds. Additionally, we study the effects of those oils as wood-biofungicides by the vapor method and confirm the activity by scanning electron microscope (SEM) examination.

## 2. Materials and Methods

### 2.1. Extraction of Essential Oils

Aerial parts of *Mentha longifolia* L. (Saudi cultivar, collected from Riyadh, Saudi Arabia, during April 2019) and leaves of *Citrus reticulata* L. (collected during March 2019 from Alexandria, Egypt) were used for the extraction. About 150 g of air-dried plant material from *M. longifolia* and another 150 g of fresh leaves from *C. reticulata* were extracted by the hydrodistillation method using a Clevenger apparatus (Local manufacturing shop, Alexandria, Egypt) [21]. The plant materials were inserted in a 2 L flask with 1.5 L of distilled water and heated for 3 h under refluxing [43]. *M. longifolia* and *C. reticulata* yielded EOs of 2.5% and 1.12%, respectively, of the dried material.

### 2.2. GC-MS Analysis of Essential Oils

The chemical constituents of the essential oils were analyzed using a Focus G C-DSQ mass spectrometer (Thermo Scientific, Austin, TX, USA). The apparatus was equipped with a direct capillary column (TG–5MS; 30 m × 0.25 mm × 0.25 µm film thickness). The initial temperature of the column oven was held at 45 °C and then increased to 200 °C at 5 °C/min and held for 5 min. The temperature was then increased to 300 °C, with 30 increments of 5 °C/min [44]. All the compounds were identified using their retention times and mass spectra by matching them with those from WILEY 09 and NIST 11 mass spectral databases. Standard Index and Reverse Standard Index measurements, with the Xcalibur 3.0 data system (3.0, Thermo Fisher Scientific Inc., Austin, TX, USA, 2014) of GC/MS, were used to confirm the identification of the compounds, where a value ≥650 is acceptable to confirm the compounds [6,7,15,16,17,18,19,20,45].

### 2.3. Fungal Isolates, the Antifungal Activity Method and the Application on Wood

Fungi of *Aspergillus flavus* AFl375, *A. niger* Ani245, *A. fumigatus* Afu694, and *Fusarium culmorum* Fcu761, with their accession numbers of MH355958, MH355955, MH355959 and MH355954, were used for the bioassay evaluation. All the fungal isolates were identified using a partial ITS gene [9]. The EOs were prepared at the concentrations of 500, 250, 125, and 65 µL/mL by dilution in 10% dimethyl sulfoxide (DMSO); a few drops of Tween80 (0.01%) were added based on potato dextrose agar (PDA) medium. Tests of inhibition of microorganisms were performed in 9 cm Petri dishes with PDA, with and without the EOs. For comparisons, the standard antibiotic of Sertaconazol 3 g/L as a positive and 10% DMSO, with Tween80 as negative control, was used. Each treatment was evaluated in triplicate. Seven-day-old colonies from each fungus, measuring 9 mm, were put in the center of the treated PDA dishes and controls and incubated at 26 ± 1 °C for 14 days. When the mycelial fungus growth completely filled the Petri dish in the control treatment (negative), the fungal mycelial inhibition growth (FMIG) percentage was calculated as follows: FMIG% = [(AC − AT)/AC] × 100, where AC and AT represent the average diameters of the fungal colony of the control and treatment, respectively [46,47]. The lowest two concentrations were used for the application on wood samples of *Pinus roxburghii Sarg*. to show their activity as wood-biofungicides using vapor treatment [48,49,50].

### 2.4. SEM Examination of Inoculated Wood

Scanning electron microscope (SEM) examination was used to show the fungal growth on *P. roxburghii* wood samples that were treated and untreated with oils and inoculated with each of the four molds using the JFC-1100E ion sputtering device (model JSM-5300, JEOL, Tokyo, Japan) at 8 kV [8,9,11,51].

### 2.5. Computation Method

Based on semiempirical calculations, geometry optimization of the studied molecules was done using the molecular modeling program Hyperchem7.5 (W.Thiel 2003, HyperChemTM, Release 7.5 Pro 2002, Athens, GA, USA). Semiempirical calculations were carried out using the routine Modified Neglect of Diatomic Overlap (MNDO) and Polak–Ribiere conjugated gradient algorithm, as shown in previous works [18,52,53].

### 2.6. Statistical Analysis

The mycelial inhibition growth percentage values were statistically analyzed for two factors (EO type and EO concentration) using analysis of variance and the Statistical Analysis Software (SAS, Release 8.02, Cary, NC, USA) system [54]. Differences among means were measured using Duncan’s multiple range test at alpha < 0.05.

## 3. Results

### 3.1. Essential Oil Composition

**Figure 1:**
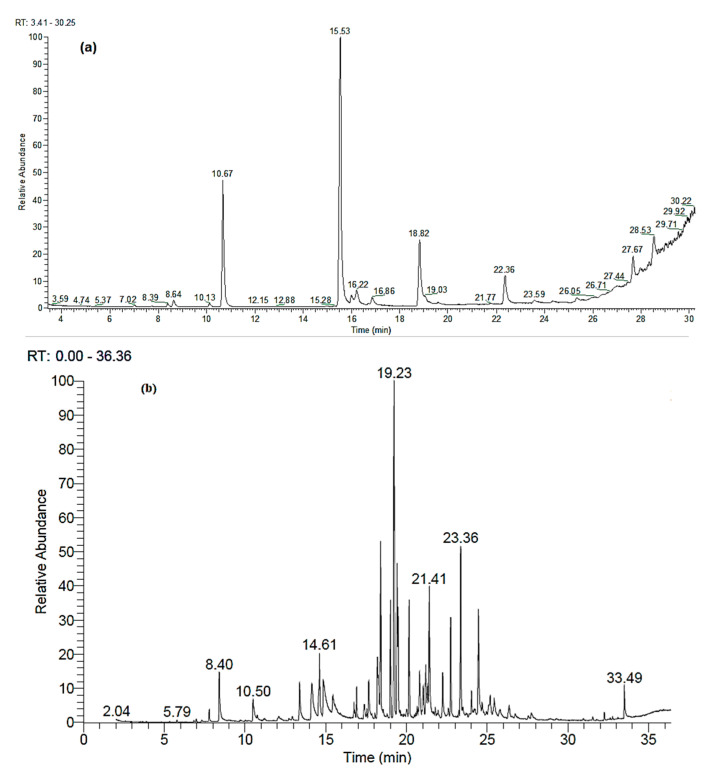
Gas chromatography–mass spectrometry (GC-MS) separation chromatograms for the essential oils from aerial parts of (**a**) *M. longifolia* and (**b**) *C. reticulata*. R.T. (Retention time, min).

Figure 1a,b shows the GC/MS chromatograms of the separated chemical compounds in the essential oils (EOs) from aerial parts of *M. longifolia* and leaves of *C. reticulata*, respectively. Table 1 presents the chemical composition of the EO from *M. longifolia* aerial parts, from which 8 compounds were identified. The main constituents were menthone (48.00%), eucalyptol (21.66%), and pulegone (12.09%). Table 2 presents the chemical compounds of the EO from *C. reticulata* aerial parts, which were composed of 48 compounds, where the main compounds were β-caryophyllene (15.57%), β-caryophyllene oxide (7.04%), β-elemene (6.39%), γ-elemene (5.62%), β-bisabolene (4.86%), spathulenol (4.74%), α-caryophyllene (4.53%), longifolene (4.40%), γ-gurjunene (3.74%), geranyl acetate (3.34%), α-bergamotene (3.19%), linalyl acetate (2.96%), germacrene D (2.28%), nerol (2.24%), d-limonene (2.14%), and geraniol (2.00%).

### 3.2. Thermodynamic Data for the Most Abundant Essential Oil Compounds

In this study, the main components in the two studied samples were divided into two groups. In the first group (Group I) were the compounds containing an oxygen atom in a single or double bond in their structures, namely, eucalyptol, menthone, pulegone, and β-caryophyllene oxide. In the second group (Group II) were the compounds containing no oxygen atom in their structures, which included β-caryophyllene and γ-elemene. Table 3 shows all thermodynamic data calculated using Modified Neglect of Diatomic Overlap (MNDO) semiempirical calculations. From the calculated data of the studied molecules (Table 3), one can observe that the negative values of the heat of formations (ΔF(M)) and total energy for Group I (eucalyptol, menthone, pulegone, and caryophyllene oxide) neutral molecules have negative values, which means these molecules are stable; the menthone molecule is the most stable. This is due to the presence of an oxygen atom (single or double bond) in their structures, while Group II β-caryophyllene and γ-elemene have positive values of heat of formations (12 and 47 Kcal/mol, respectively). From these values, the second group is relatively less stable than the first group, which has oxygen atoms in the structure of its compounds. This is confirmed by the values of dipole moment; hence, the first group has higher values of dipole moment (1.384, 2.446, 2.497, and 1.684) in comparison with the second group (0.133 and 0.158). In addition, one can observe that Group I has higher values of ionization energies (9.4, 9.5, 9.0, and 8.8 eV) in comparison with Group II (8.7 and 8.8 eV). These due to the higher stability of Group I. The same was observed for electron affinity values: Group I had higher values of EA, as shown in Table 3.

### 3.3. Antifungal Activity of the Essential Oils

The inhibition of fungal mycelia correlates positively with concentration (Figures S1–S4).

Table 4 shows the antifungal activity of EOs from *M. longifolia* and *C. reticulata* against the growth of *A. flavus*, *A. niger*, *A. terreus*, and *F. culmorum*. For *M. longifolia* and *C. reticulata* EOs, at the concentrations of 500 and 250 µL/mL, antifungal activity was observed against *A. flavus* and *A. fumigatus*, with 100% fungal mycelial inhibition growth (FMIG), which was higher than the FMIG values from Sertaconazol (91% and 88.66%, respectively). *C. reticulata* and *M. longifolia* EOs, at 125 µL/mL, showed activity against the growth of *A. fumigatus*, with FMIG values of 98% and 95%, respectively. *M. longifolia* EO, at 500 and 250 µL/mL, showed 100% FMIG against *A. niger*, while *C. reticulata* EO showed FMIG values of 100% and 97%, at 500 and 250 µL/mL, respectively, which were higher than the value from Sertaconazol (87%). The EOs from *M. longifolia* and *C. reticulata* completely inhibited the growth of *F. culmorum*, with 100% FMIG at the concentration of 500 µL/mL, which is higher than the value from Sertaconazol (88.33%). Additionally, at the concentration of 250 µL/mL, the EO from *C. reticulata* showed activity against *F. culmorum* (FMIG value of 85.66%). Furthermore, EOs at lower concentrations (65 and 125 µL/mL) showed FMIG percentages against the studied molds. Therefore, those two concentrations were used for the application on wood by the vapor method.

### 3.4. Application of Oils on Wood Samples

**Figure 2:**
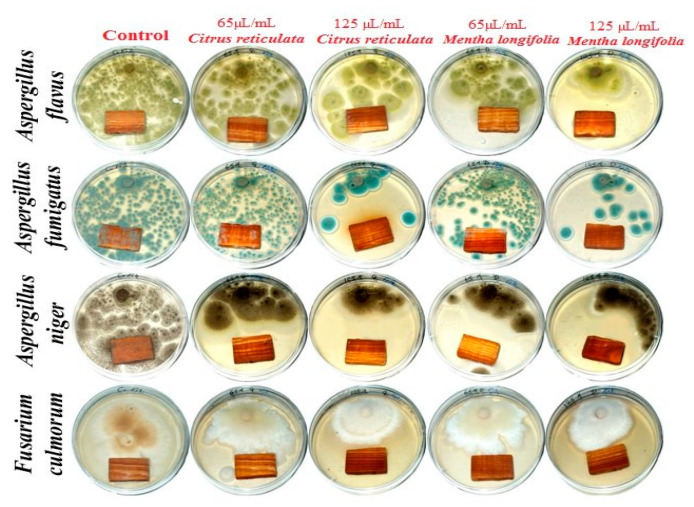
Experimental application of essential oils from *Mentha longifolia* and *Citrus reticulata* to *Pinus halepensis* wood and the visual observation of growth of *A. flavus*, *A. niger*, *A. terreus*, and *F. culmorum*.

Figure 2 shows that both oils from *M. longifolia* and *C. reticulata*, at 125 µL/mL, showed inhibition zone (IZ) values around the treated *Pinus roxburghii* wood samples compared to the control treatment (wood without oils), where the growth of fungi was observed. Table 5 presents the antifungal activity of both oils as wood-biofungicides by measuring the growth on wood samples and the IZs around the wood. *M. longifolia*, applied to wood at 125 µL/mL, showed an IZ value of 7.33 mm against the growth of *A. flavus*. An IZ value of 15.33 mm was shown for wood treated with *C. reticulata* oil at 125 µL/mL against *A. fumigatus*. *M. longifolia*, at 65 and 125 µL/mL, showed potent antifungal activity against *A. niger* when applied to wood samples with IZ values of 8 and 21.33 mm, respectively. Wood treated with *C. reticulata* and *M. longifolia*, at 125 µL/mL, showed IZ values of 3.66 and 2.33 mm, respectively, against the growth of *F. culmorum*.

### 3.5. SEM Examination of Inoculated Wood with Fungi

**Figure 3:**
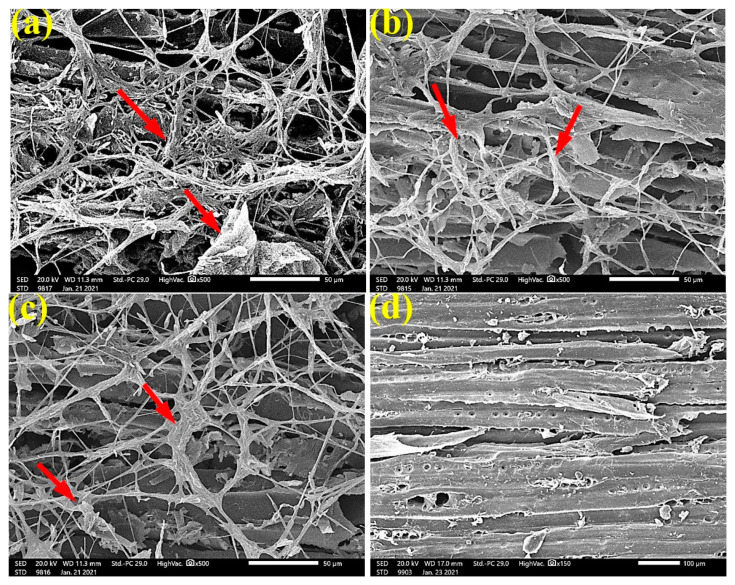
SEM images of *P. roxburghii* wood samples inoculated with *F. culmorum*: (**a**,**b**) without treatment; (**c**) with 65 µL/mL *C. reticulata* EO; (**d**) with 125 µL/mL *C. reticulata* EO. Arrows refer to the growth of fungal mycelia in wood samples, according to treatment.

As shown in the SEM images (Figure 3), fungal mycelia growth (FMG) of *F. culmorum* was observed over *P. roxburghii* wood samples without treatment (Figure 3a,b) and treated with 65 µL/mL *C. reticulata* EO (Figure 3c). In contrast, FMG was inhibited or suppressed when the wood was treated with 125 µL/mL *C. reticulata* EO (Figure 3d), where the wood structures, such as tracheids and pits, are clearly shown with no fungal biomass.

**Figure 4:**
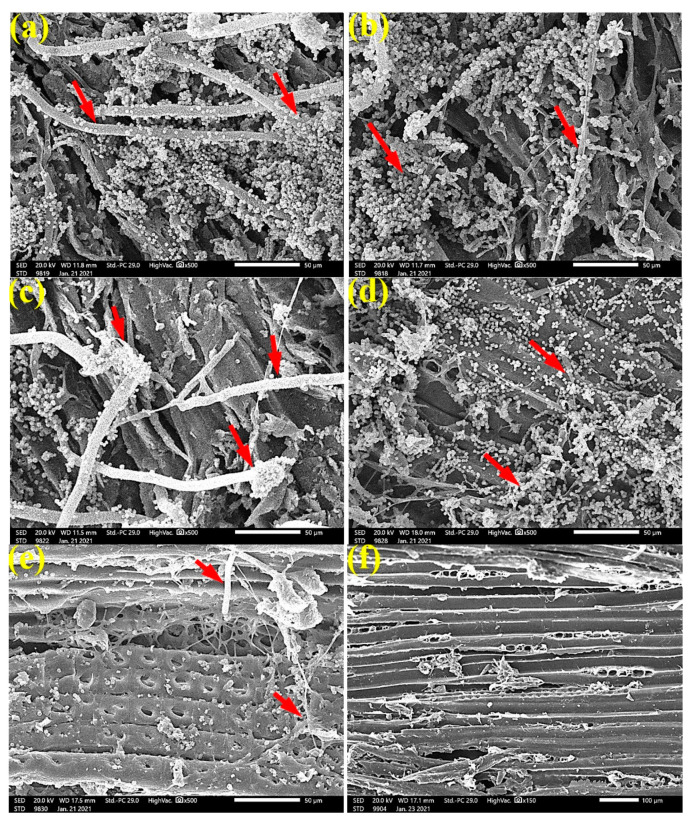
SEM images of *P. roxburghii* wood samples inoculated with *A. flavus*: (**a**) without treatment; (**b**) with 125 µL/mL *C. reticulata* EO; (**c**) with 65 µL/mL *M. longifolia* EO; (**d**) with 125 µL/mL *M. longifolia* EO; (**e**) with 65 µL/mL *M. longifolia* EO; (**f**) with 125 µL/mL *M. longifolia* EO. Arrows refer to the dense growth of fungal mycelia in wood samples, according to treatment.

SEM images in Figure 4 clearly show the growth of *A. flavus* over *P. roxburghii* wood samples without treatment (Figure 4a), treated with 125 µL/mL of *C. reticulata* EO (Figure 4b), treated with 65 µL/mL of *M. longifolia* EO (Figure 4c), and treated with 125 µL/mL *M. longifolia* EO (Figure 4d). FMG was reduced when *P. roxburghii* wood samples were treated with 65 µL/mL *M. longifolia* EO (Figure 4e), and FMG was inhibited when the wood samples were treated with 125 µL/mL *M. longifolia* EO (Figure 4f), where tracheids and wood rays are clearly shown.

**Figure 5:**
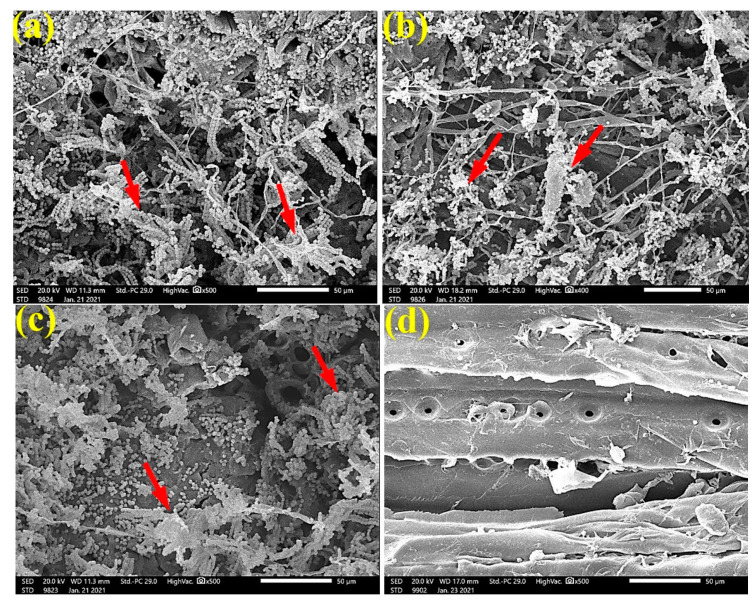
SEM images of P. roxburghii wood samples inoculated with Aspergillus fumigatus: (**a**) without treatment; (**b**) with 65 µL/mL C. reticulata EO; (**c**) with 65 µL/mL M. longifolia EO; (**d**) with 125 µL/mL M. longifolia EO. Arrows refer to the dense growth of fungal mycelia in wood samples, according to treatment.

Additionally, in Figure 5, the SEM images of *P. roxburghii* wood samples inoculated with *A. fumigatus* showed a high level of fungal growth without treatment (Figure 5a), with 65 µL/mL *C. reticulata* EO (Figure 5b), and with 65 µL/mL *M. longifolia* EO (Figure 5c). Significantly, the wood treated with 125 µL/mL *M. longifolia* EO showed complete inhibition of *A. fumigatus* fungal growth (Figure 5d); the anatomical features from tracheids and bordered pits are clearly shown.

**Figure 6:**
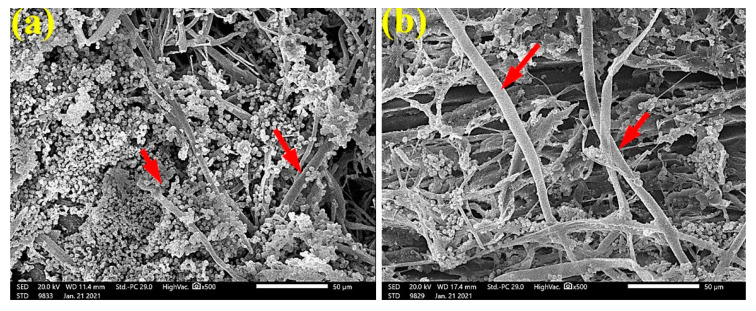

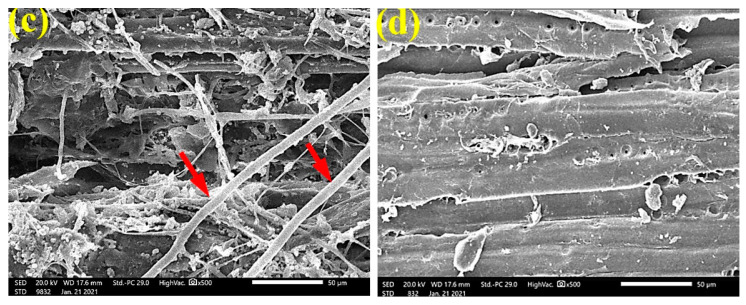
SEM images of *P. roxburghii* wood samples inoculated with *A. niger*: (**a**,**b**) without treatment; (**c**) with 65 µL/mL *M. longifolia* EO; (**d**) with 125 µL/mL *M. longifolia* EO. Arrows refer to the dense growth of fungal mycelia in wood samples, according to treatment.

SEM images in Figure 6 clearly show the high level of growth of *A. niger* over *P. roxburghii* wood samples without treatment (Figure 6a,b) and with 65 µL/mL *M. longifolia* EO (Figure 6c); significant reduction and inhibition of fungal growth were observed when wood samples were treated with 125 µL/mL *M. longifolia* EO (Figure 6d), where pits and tracheids are shown without fungal penetrations.

## 4. Discussion

*M. longifolia* aerial part EO shows the presence of menthone, eucalyptol (1,8-cineole), and pulegone as the main compounds. Chemical composition of the EO of *M. longifolia* grown around the world has shown different chymotypes. Menthone and iso-menthone have been found in the range amounts of 2.8–15.05% and 0.96–43.79%, respectively, from the *M. longifolia* plants grown in Iran-Asia [55]. Menthone (10.7%), pulegone (47.15%), and 1,8-cineole (11.54%) have been found in *M. longifolia* plants grown in Tunisia [56], and menthol (19.4–32.5%), menthone (20.7–28.8%), 1,8-cineole (5.6–10.8%), terpineol-4 (3.1–4.9%), and pulegone (7.8–17.8%) have been found in *M. longifolia* plants grown in Southern Africa [29]. In addition, in *M. longifolia* plants grown in Serbia, menthone (11.2%) and piperitone (8.8%) were identified as the main compounds [35]. The main compounds from *M. longifolia* plants growing in South Africa were menthone, eucalyptol, and pulegone [57]. Piperitone is the major compound (30.77%), followed by eucalyptol (14.85%) and caryophellene (5.58%), in the EO of leaves from Saudi *M. longifolia* [37], while Desam et al. [58] reported that menthone (39.55%), isopulegone (30.49%), eucalyptol (10.38%), and α-terpineol (3.15%) were major components from *M. longifolia* aerial parts that were air-dried under shade, with promising antibacterial activity against *Staphylococcus aureus*, *Enterococcus faecalis*, and *Bacillus cereus* and antifungal activity against *Aspergillus flavus*, *A. fumigates*, *Alternaria alternaria*, *Fusarium oxyporum*, and *Penicillum* spp.

Eucalyptol, found at 21.66% in *M. longifolia* EO, was also reported to be one of the major compounds in Mentha species EO, which ranged from 1.6% to 15.58% in plants from Iran [59,60,61], Tunisia [54,62] and Italy [63]. Pulegone, eucalyptol, and L-menthone, with percentages of 26.92%, 21.3%, and 10.66%, respectively, were found as the main compounds in the EO of plants grown in the winter season, while the main compounds found in the EO from the plants grown in spring were pulegone, oleic acid, and palmitic acid, with percentages of 38.2%, 23.79%, and 15.26%, respectively [32].

Other chymotypes were piperitenone oxide and piperitone oxide in plants growing in the Mediterranean region [27,60,63,64,65]. The *M. longifolia* plants growing in Iran [66] and Sudan [67] are rich in carvone, while the EOs from Jordan [65] and Tunisia [62] are rich in pulegone. Additionally, plants grown in Iran contain a eucalyptol-rich chemotype [59] or are rich in carveol [68]. *M. longifolia* EO from plants grown in Croatia showed the presence of piperitenone oxide, β-Caryophyllene, carvone, and limonene as the main compounds [69]. The EO from *M. longifolia* flowers collected from Zlatar, Belgrade, Serbia, showed the presence of *trans*- and *cis*-dihydrocarvone, piperitone, eucalyptol, and neoisodihydrocarveol as the main compounds, with 23.64%, 15.68%, 17.33%, 8.18%, and 7.87%, respectively [31]. Piperitone oxide, in high amounts, and piperitenon oxide were found in *M. longifolia* EO from Morocco [70], while *cis*-carveol was the dominant compound in *M. longifolia* from Iran, with percentages ranging from 53% to 78% [68]. Piperitone oxide and piperitenone oxide were found as the main compounds of the EO from the plants grown in Turkey [71]. From Iran, they were piperitone, limonene, and *trans*-piperitol [72]; from France, they were carvone, 1,8-cineole, and limonene [73]. The EO of *M. longifolia* growing wild in the Bahcesaray area (Van Province, Turkey) showed the presence of menthone (19.31%), pulegone (12.42%), piperitone (11.05%), dihydrocarvon (8.32%), limonene (6.1%), 3-terpinolenone (5.66%), eucalyptol (4.37%), germacrene D (3.38%), and caryopyllene (3.19%) as the main components [74].

The plants grown in India, in different locations, showed the presence of piperitenone oxide, *cis/trans*-piperitone oxide, eucalyptol, piperitenone, dl-limonene, piperitone, 4-hydroxy piperitone, and β-caryophyllene [59,75,76,77,78,79]. EOs, with their main compounds (carvone, limonene, and 1,6-dihydrocarveol) from plants grown in five regions (Saudi Arabia), showed moderate antifungal activity against *A. niger*, *A. flavus*, and *F. solani* [28]. At 250 ppm, the EO of *M. longifolia* inhibited the growth of *F. oxysporum* (92%), followed by *Sclerotuim rolfsii* (70.66%) and *Rhizoctonia solani* (57.04%) [75]. The EO from *M. longifolia*, at 10 μL/mL, showed potent fungicidal activity against *A. niger*, *A. ochraceus*, *A. flavus*, *A. versicolor*, *F. tricinctum*, *F. sporotrichioides*, *Penicillium funiculosum* and *Trichoderma viride* [31].

Some previously reported bioactive compounds were found in the EO from *C. reticulata*, such as β-caryophyllene, β-caryophyllene oxide, β-elemene, γ-elemene, β-bisabolene, spathulenol, α-caryophyllene, longifolene, γ-gurjunene, geranyl acetate, α-bergamotene, linalyl acetate, germacrene D, nerol, d-limonene, and geraniol. Among some citrus EOs (*Citrus lemon*, *C. reticulata*, *C. paradisi* and *C. sinensis*), the EO from *C. reticulata* showed the lowest activity against *Lactobacillus curvatus*, *L. sakei*, *Staphylococcus carnosus*, *S. xylosus*, *Enterobacter gergoviae* and *E. amnigenus* [22]. Hydrocarbons and linalool were mostly found in leaf EO from *C. reticulata*, while thymol and/or terpinen-4-ol were found in leaf EO of some varieties [80,81]. In 41 mandarin cultivars, γ-terpinene, sabinene, linalool, limonene, *p*-cymene, (*E*)-β-ocimene, β-pinene, and terpinen-4-ol were found in the range of 0.2–61.3%, 0.2–59.4%, 0.2–54.3%, 1.5–44.3%, traces–20.4%, 0.6–13.7%, 0.1–10.7%, and 0.1–10.6%, respectively, in the EO from leaves [40].

The EO from the peel of fully matured ripen fruits of *C. reticulata* Blanco, with its main compounds of limonene, geranial, neral, geranyl acetate, geraniol, β-caryophyllene, nerol, and neryl acetate) showed good activity against some pathogenic fungi, namely, *Alternaria alternata*, *Rhizoctonia solani*, *Curvularia lunata*, *F. oxysporum*, and *Helminthosporium oryzae* [41]. Mature fruit EO of *C. reticulata* showed the presence of citronellol, octanal, decanal, nonanal, β-pinene, limonene, citral, γ-terpinene, linalool, and α-terpineol, with high antifungal activity against the growth of *Penicillium italicum* and *P. digitatum* [82]. Leaf EO from six cultivars of *C. reticulata* Blanco from Nigeria showed the presence of sabinene, γ-terpinene, *p*-cymene, δ-3-carene, and (*E*)-β-ocimene, while other constituents include linalool, myrcene, terpinen-4-ol, and *cis*-sabinenehydrate. In addition, limonene, terpinolene, β-pinene, α-pinene, β-sinensal, and α-sinensal were detected and isolated [38]. Geranial, neryl acetate, geranyl acetate, β-pinene, myrcene, neral, and β-caryophyllene have been identified in the leaf EO of *C. limon* [83].

The applied EOs from *M. longifolia* and *C. reticulata* to *Pinus roxburghii* wood showed good activity against the growth of *A. flavus, A. niger*, *A. terreus* and *F. culmorum*. Previously, EOs and extracts have been used as wood-biofungicides and have shown some potential antifungal activity, i.e., *Acacia saligna* wood treated with methanol extract from *Maclura pomifera* bark against *Alternaria tenuissima* [2] and *A. saligna* wood treated with *Cupressus sempervirens* methanolic extract against *Trichoderma harzianum* infestation [3]. Wood samples from *P. sylvestris*, *P. rigida* and *Fagus sylvatica*, treated with two EOs of *P. rigida* (wood) and *Eucalyptus camaldulensis* (leaves), showed promising antifungal activity against five molds (*A. alternata*, *F. subglutinans*, *C. globosum*, *A. niger* and *T. viride*) [50]. Wood samples from *A. saligna, F. sylvatica, Juglans nigra* and *P. rigida*, treated with the oil from *Origanum majorana* leaves, showed good antifungal activity against *T. harzianum* and *A. niger* without changing the wood structures [17]. *Leucaena leucocephala* wood, treated with *Acer saccharum var. saccharum* extract from inner or outer bark, in combination with citric acid, showed bioactivity against the growth of *T. viride*, *F. subglutinans* and *A. niger* [6]. *Melia azedarach* wood, treated with *E. camaldulensis* or *V. agenus-castus n*-hexane oily extracts, showed potential antifungal against *F. culmorum*, *R. solani* and *P. chrysogenum* [7]. *M. azedarach* wood samples, treated with *A. saligna* flower extract, showed promising antifungal activity against *P. chrysogenum* [4]. *M. azedarach* wood treated with *Musa paradisiaca* extract showed good bioactivity against *F. culmorum* and *R. solani* [5]. Chinaberry wood blocks, treated with *E. camaldulensis* bark extract, showed potential antifungal activity against *F. culmorum* and *Botrytis cinerea* [84], and *M. azedarach* wood treated with whole-plant extract of *Haplophyllum tuberculatum* showed good antifungal activity against *F. culmorum* and *R. solani* [85]. *Corymbia citriodora* EOs from leaves (the main compounds were citronellal, citronellol, and isopulegol) and fruits (α-pinene, eudesmol, limonene, γ-terpinene as main compounds), applied to wood samples at the amounts of 100, 50 and 25 µL, showed 100% inhibition against *F. culmorum* [18]. Recovered oil dissolved in *n*-hexane solvent, as partitioned from the distillate residue of hydrodistillation of fresh flowers from *Matricaria chamomilla*, showed potent bioactivity activity against *A. niger* and *A. terreus* [43].

To ensure the complete inhibition of fungal growth, SEM examinations showed a clear inhibition of the four studied fungi with the application oils at the concentration of 125 µL/mL. The EOs might alter the hyphal morphology. Indeed, it can be seen from the SEM images that the fruiting bodies (conidiophores) are lower in number or do not exist, similar for spores, and the hyphae are altered.

## 5. Conclusions

The findings of the present research confirmed the potent antifungal activities of essential oils from *Mentha longifolia* (Saudi cultivar) and *Citrus reticulata* (Egyptian growing plant). Essential oils from *M. longifolia* and *C. reticulata* are of great interest with regard to their antifungal activities against *Aspergillus flavus*, *A. niger*, *A. fumigatus*, and *Fusarium culmorum* when applied as biopreservation for wood. Essential oil from *M. longifolia*, at 125 µL/mL, applied to *Pinus roxburghii* wood, showed an inhibition zone (7.33 mm) around the wood samples when inoculated with *A. flavus*; the inhibition zone was 15.33 mm when the wood samples were treated with the essential oil from *C. reticulata* against *A. fumigatus* and 21.33 mm when treated with the oil from *M. longifolia* against *A. niger*. Additionally, both oils showed the lowest inhibition zones against *F. culmorum* when applied to wood samples. By SEM examination, wood anatomical features have been clearly shown to have no fungal growths when the wood samples were treated with both oils at 125 µL/mL. These activities suggest their possible use as natural preservative additives and in the food industry.

## Figures and Tables

**Table 1:** Chemical composition of the essential oil from *Mentha longifolia* aerial parts.

Compound Name	Percentage in the Oil (%)
Eucalyptol or 1,8-cineole	21.66 (946-947) *
Menthone	48.00 (946-969)
Borneol	2.10 (885-910)
Pulegone	12.09 (917-934)
β-Caryophyllene	5.57 (910-938)
2-Methylene-5α-cholestan-3β-ol	4.89 (812-845)
1-Heptatriacotanol	5.69 (767-777)
Oxygenated Monoterpenes	83.85
Sesquiterpenes	5.57
Pentacyclic triterpenes	4.89
Fatty alcohol (%)	5.69

* Values in parentheses are Standard Index (SI) and Reverse Standard Index (RSI).

**Table 2:** Chemical composition of the essential oil from *Citrus reticulata* leaves.

Compound Name	Percentage in the Oil (%)
Δ-3-Carene	0.41 (922-937) *
d-Limonene	2.14 (923-925)
Linalool	1.12 (943-954)
Citronellal	0.50 (883-902)
Terpinen-4-ol	0.17 (879-882)
α-Terpineol	1.70 (938-940)
Nerol	2.24 (862-887)
Linalyl acetate	2.96 (885-889)
Geraniol	2.00 (929-935)
Citral	1.41 (888-901)
Δ-Elemene	1.65 (854-897)
Isopulegol acetate	0.89 (782-843)
γ-Muurolene	0.24 (836-855)
Neryl acetate	1.63 (926-932)
α-Himachalene	0.17 (870-887)
Geranyl acetate	3.34 (902-936)
β-Elemene	6.39 (934-938)
Longifolene	4.40 (958-958)
β-Caryophyllene	15.57 (945-946)
γ-Elemene	5.62 (945-947)
α-Bergamotene	3.19 (933-955)
Nerolidol	0.40 (760-777)
α-Caryophyllene	4.53 (935-945)
Ylangene	0.23 (838-862)
Germacrene D	2.28 (924-935)
β-Selinene	1.34 (928-959)
α-Selinene	1.88 (943-958)
α-Farnesene	0.69 (917-945)
β-Bisabolene	4.86 (928-942)
Selina-3,7(11)-diene	0.25 (859-881)
*cis*-*Z*-α-Bisabolene epoxide	0.36 (810-817)
*trans*-Longipinocarveol	1.86 (802-810)
Caryophyllene oxide	0.43 (856-901)
γ-Gurjunene	3.74 (895-911)
β-Caryophyllene oxide	7.04 (954-956)
Isoaromadendrene epoxide	0.17 (798-847)
Humulene oxide II	0.85 (818-901)
Alloaromadendrene	0.12 (799-860)
Nerolidol-epoxyacetate	0.17 (799-853)
Spathulenol	4.74 (883-884)
Globulol	1.46 (839-871)
Guaiene	0.91 (831-842)
2-Methylene-5α-cholestan-3β-ol	0.47 (789-811)
Ledene oxide-(II)	0.34 (824-842)
Squalene	0.30 (708-716)
Urs-12-en-28-ol	1.13 (736-787)
Monoterpene hydrocarbons	2.59
Oxygenated monoterpenes	18.27
Sesquiterpene hydrocarbons	59.07
Oxygenated sesquiterpenes	18.13
Pentacyclic triterpenes	1.62
Triterpene hydrocarbon	0.30

* Values in parentheses are Standard Index (SI) and Reverse Standard Index (RSI).

**Table 3:** Thermodynamic data of the studied molecules, calculated within the modified neglect of the diatomic overlap (MNDO) framework.

Compound	Total Energy (TE) (Kcal/mol)	ΔH_f_[M] (Kcal/mol)	ΔH_f_[M]^+•^ (Kcal/mol)	Δ_f_[M]^−1^ (Kcal/mol)	Dipole Moment (Debye)	Ionization Energy (IE) * eV	Electron Affinity (EA) ** eV
Eucalyptol	−42,819	−52	166	−31	1.384	9.4	0.91
Menthone	−42,831	−64	156	−67	2.446	9.5	0.13
Pulegone	−42,164	−50	156	−71	2.497	9.0	0.91
β-Caryophyllene oxide	−59,481	−5	199	−3	1.684	8.8	0.08
β-Caryophyllene	−52,074	12	213	11	0.133	8.7	0.04
γ-Elemene	−52,039	47	252	49	0.158	8.8	0.08

* The values of ionization energies (IE) were calculated with the following equation: IE [M] = ΔH_f_ [M]^+•^ − ΔH_f_ [M], where ΔH_f_ [M]^+•^ and ΔH_f_ [M] are the heat of formation of the molecular ion and neutral molecule, respectively. ** The values of the electron affinity (EA) were calculated with the following equation: EA [M] =ΔH_f_ [M] − ΔH_f_ [M]^−1^, where ΔH_f_ [M]^−1^ and ΔH_f_ [M] are the heat of formation of the anion and neutral molecule, respectively.

**Table 4:** Inhibition percentage of the diameter growth of *A. flavus*, *A. niger*, *A. terreus*, and *F. culmorum*, as affected by essential oils (EOs) from *Mentha longifolia* and *Citrus reticulata*.

Oil Source	Concentration (µL/mL)	Inhibition Percentage of Diameter Growth (%)			
		*Aspergillus* *flavus*	*Aspergillus* *fumigatus*	*Aspergillus* *niger*	*Fusarium* *culmorum*
*Mentha longifolia*	65	48 ± 3.46	70 ± 2.64	63 ± 2	46.66 ± 1.15
	125	74.33 ± 1.52	95 ± 1	73.33 ± 1.52	70 ± 1
	250	100	100	100	73.33 ± 3.21
	500	100	100	100	100
*Citrus reticulata*	65	86.33 ± 0.57	68.66 ± 3.05	65.33 ± 1.52	65.66 ± 1.15
	125	91 ± 3.61	98 ± 3.46	93.66 ± 0.57	81 ± 3
	250	100	100	97 ± 2.64	85.66 ± 0.57
	500	100	100	100	100
Negative control (DMSO)	10%	0.00	0.00	0.00	0.00
Sertaconazol	3 g/L	91 ± 1	88.66 ± 1.15	87 ± 1	88.33 ± 1.52
*p*-value		**	**	**	**

Values are means ± SE. ** Highly significant at 0.01 level of probability.

**Table 5:** Growth on wood samples ** and inhibition zones ** (mm) of *A. flavus*, *A. niger*, *A. terreus*, and *F. culmorum*, as affected by essential oils from *Mentha longifolia* and *Citrus reticulate*.

Oil Source	Concentration µL/mL	*Aspergillus flavus*		*Aspergillus fumigatus*		*Aspergillus niger*		*Fusarium culmorum*	
		Growth on Sample (mm)	IZ (mm)	Growth on Sample (mm)	IZ (mm)	Growth on Sample (mm)	IZ (mm)	Growth on Sample (mm)	IZ (mm)
Control	0	20 ± 0.00	0.00	20	0.00	20	0.00	18.33 ± 0.88	0.00
*C. reticulata*	65	0.00	0.33 ± 0.33	5.66 ± 1.20	0.00	0.00	2.33 ± 0.33	0.00	1 ± 0.57
	125	0.00	0.66 ± 0.33	0.00	15.33 ± 3.75	0.00	5.33 ± 0.88	0.00	3.66 ± 0.88
*M. longifolia*	65	0.00	0.66 ± 0.33	2 ± 0.57	0.00	0.00	8.00 ± 2.00	0.00	1 ± 0.57
	125	0.00	7.33 ± 1.20	0.0000	3.33 ± 0.88	0.00	21.33 ± 5.69	0.00	2.33 ± 1.45
*p*-value		**	**	**	**	**	**	**	**

Values are means ± SE. ** Highly significant at 0.01 level of probability.

## Data Availability

The data presented in this study are available on request from the corresponding author.

## Supplementary Materials

The following are available online at https://www.mdpi.com/1996-1944/14/6/1361/s1, Figure S1: Visual observation of *Aspergillus flavus* growth inhibition as affected by the EOs from (A) *Mentha longifolia*; (B) *Citrus reticulata*; (P) Positive control (Sertaconazol 3 g/L); (N) Negative control (DMSO 10%), Figure S2: Visual observation of *Aspergillus niger* growth inhibition as affected by the EOs from (A) *Mentha longifolia*; (B) *Citrus reticulata*; (P) Positive control (Sertaconazol 3 g/L); (N) Negative control (DMSO 10%), Figure S3: Visual observation of *Aspergillus fumigatus* growth inhibition as affected by the EOs from (A) *Mentha longifolia*; (B) *Citrus reticulata*; (P) Positive control (Sertaconazol 3 g/L); (N) Negative control (DMSO 10%), Figure S4: Visual observation of *Fusarium culmorum* growth inhibition as affected by the EOs from (A) *Mentha longifolia*; (B) *Citrus reticulata*; (P) Positive control (Sertaconazol 3 g/L); (N) Negative control (DMSO 10%).
