# Supplementary material for: New Approach for Using of Mentha longifolia L. and Citrus reticulata L. Essential Oils as Wood-Biofungicides: GC-MS, SEM, and MNDO Quantum Chemical Studies
Source: Materials (Basel). 2021 Mar 11;14(6):1361. doi: 10.3390/ma14061361 (PMC7998113; doi:10.3390/ma14061361)
Supplement: Supplementary file 1 [file materials-14-01361-s001.pdf]

materials

Article

New Approach for Using of *Mentha longifolia* L. and *Citrus reticulata* L. Essential Oils as Wood-Biofungicides: GC-MS, SEM, and MNDO Quantum Chemical Studies

Hayssam M. Ali [1,2], Wael A.A. Abo Elgat [3], Mervat EL-Hefny [4], Mohamed Z.M. Salem [5,*], Ayman S. Taha [6], Dunia A. Al Farraj [1], Mohamed S. Elshikh [1], Ashraf A. Hatamleh [1] and Eslam M. Abdel-Salam [1]

1 Botany and Microbiology Department, College of Science, King Saud University, P.O. Box 2455, Riyadh 11451, Saudi Arabia; hayhassan@ksu.edu.sa (H.M.A.); dfarraj@ksu.edu.sa (D.A.A.F.); melshikh@ksu.edu.sa (M.S.E.); ahatamleh@ksu.edu.sa (A.A.H.); 436108002@student.ksu.edu.sa (E.M.A.-S.)
2 Agriculture Research Center, Timber Trees Research Department, Sabahia Horticulture Research Station, Horticulture Research Institute, Alexandria 21526, Egypt
3 Restoration Department, High Institute of Tourism, Hotel Management and Restoration, Abukir, Alexandria 21526, Egypt; watsat20@yahoo.com
4 Department of Floriculture, Ornamental Horticulture and Garden Design, Faculty of Agriculture (El-Shatby), Alexandria University, Alexandria 21545, Egypt; mervat.mohamed@alexu.edu.eg
5 Forestry and Wood Technology Department, Faculty of Agriculture (EL-Shatby), Alexandria University, Alexandria 21545, Egypt
6 Conservation Department, Faculty of Archaeology, Aswan University, Aswan 81528, Egypt; aymansalahtaha82@yahoo.com
* Correspondence: mohamed-salem@alexu.edu.eg

Citation: Ali, H.M.; Abo Elgat, W.A.A.; EL-Hefny, M.; Salem, M.Z.M.; Taha, A.S.; Al Farraj, D.A.; Elshikh, M.S.; Hatamleh, A.A.; Abdel-Salam, E.M. New Approach for Using of *Mentha longifolia* L. and *Citrus reticulata* L. Essential Oils as Wood-Biofungicides: GC-MS, SEM and MNDO Quantum Chemical Studies. *Materials* **2021**, *14*, 1361. https://doi.org/10.3390/ma14061361

Publisher's Note: MDPI stays neutral with regard to jurisdictional claims in published maps and institutional affiliations.

Copyright: © 2021 by the authors. Licensee MDPI, Basel, Switzerland. This article is an open access article distributed under the terms and conditions of the Creative Commons Attribution (CC BY) license (http://creativecommons.org/licenses/by/4.0/).

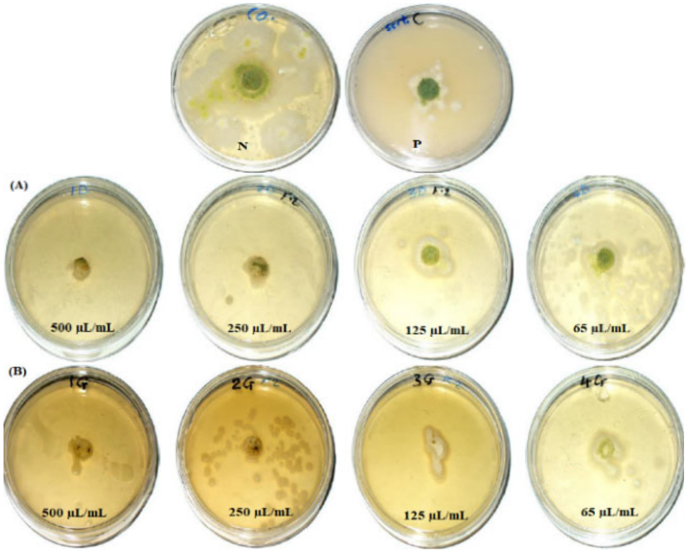

Figure S1. Visual observation of *Aspergillus flavus* growth inhibition as affected by the EOs from (**A**) *Mentha longifolia*; (**B**) *Citrus reticulata*; (P) Positive control (Sertaconazol 3 g/L); (N) Negative control (DMSO 10%).

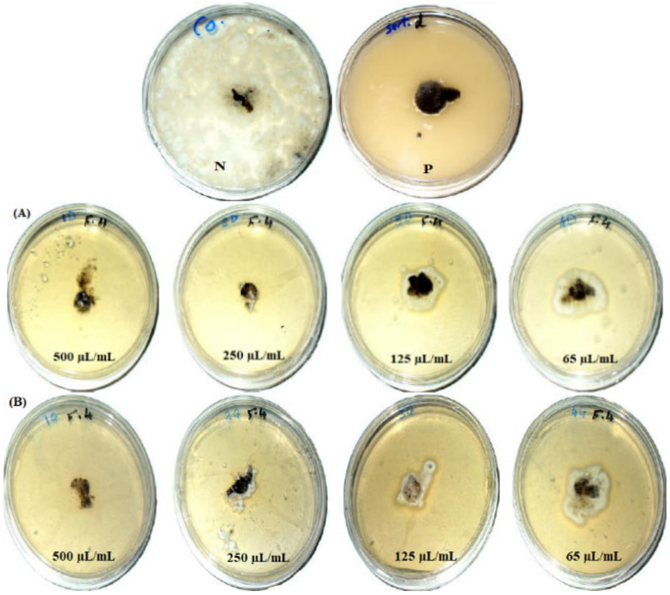

Figure S2. Visual observation of *Aspergillus niger* growth inhibition as affected by the EOs from (**A**) *Mentha longifolia*; (**B**) *Citrus reticulata*; (P) Positive control (Sertaconazol 3 g/L); (N) Negative control (DMSO 10%).

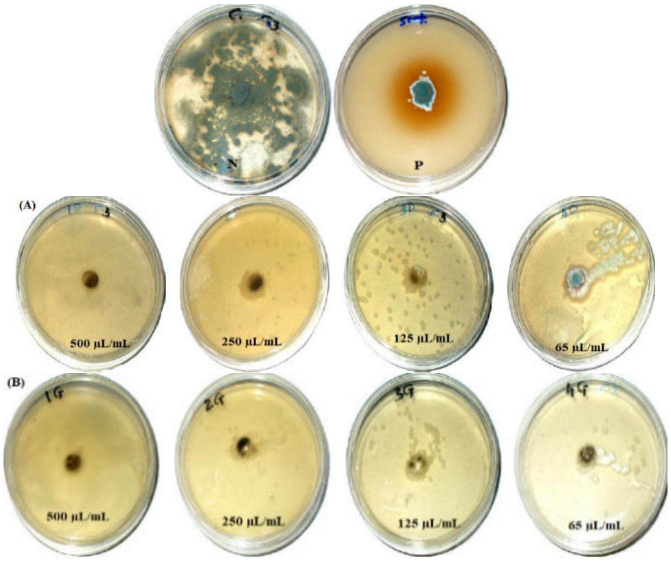

Figure S3. Visual observation of *Aspergillus fumigatus* growth inhibition as affected by the EOs from (**A**) *Mentha longifolia*; (**B**) *Citrus reticulata*; (P) Positive control (Sertaconazol 3 g/L); (N) Negative control (DMSO 10%).

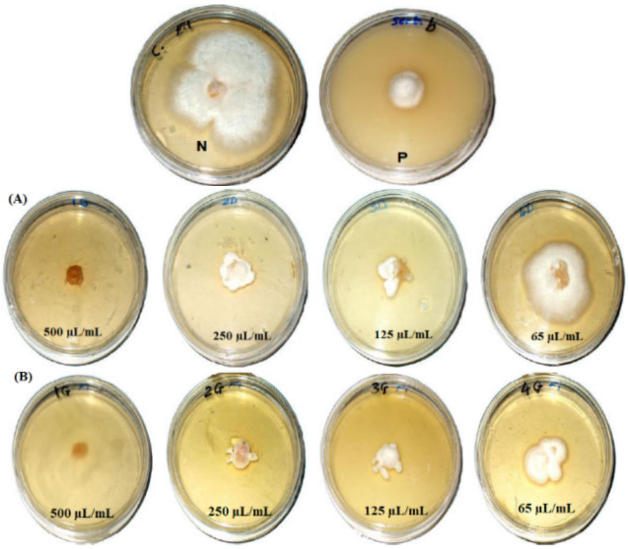

Figure S4. Visual observation of *Fusarium culmorum* growth inhibition as affected by the EOs from (**A**) *Mentha longifolia*; (**B**) *Citrus reticulata*; (P) Positive control (Sertaconazol 3 g/L); (N) Negative control (DMSO 10%).
